# Action Real-Time Strategy Gaming Experience Related to Increased Attentional Resources: An Attentional Blink Study

**DOI:** 10.3389/fnhum.2020.00101

**Published:** 2020-04-10

**Authors:** Xianyang Gan, Yutong Yao, Hui Liu, Xin Zong, Ruifang Cui, Nan Qiu, Jiaxin Xie, Dong Jiang, Shaofei Ying, Xingfeng Tang, Li Dong, Diankun Gong, Weiyi Ma, Tiejun Liu

**Affiliations:** ^1^The Clinical Hospital of Chengdu Brain Science Institute, MOE Key Lab for Neuroinformation, University of Electronic Science and Technology of China, Chengdu, China; ^2^Center for Information in Medicine, School of Life Science and Technology, University of Electronic Science and Technology of China, Chengdu, China; ^3^Faculty of Natural Science, University of Stirling, Stirling, United Kingdom; ^4^Education Center for Students Cultural Qualities, University of Electronic Science and Technology of China, Chengdu, China; ^5^School of Human Environmental Sciences, University of Arkansas, Fayetteville, AR, United States

**Keywords:** action real-time strategy gaming, visual selective attention, temporal characteristics, attentional resources, event related potentials (ERP), P3

## Abstract

Action real-time strategy gaming (ARSG) is a cognitively demanding task which requires attention, sensorimotor skills, team cooperation, and strategy-making abilities. A recent study found that ARSG experts had superior visual selective attention (VSA) for detecting the location of a moving object that could appear in one of 24 different peripheral locations (Qiu et al., 2018), suggesting that ARSG experience is related to improvements in the spatial component of VSA. However, the influence of ARSG experience on the temporal component of VSA—the detection of an item among a sequence of items presented consecutively and quickly at a single location—still remains understudied. Using behavioral and electrophysiological measures, this study examined whether ARSG experts had superior temporal VSA performance compared to non-experts in an attentional blink (AB) task, which is typically used to examine temporal VSA. The results showed that the experts outperformed the non-experts in their detection rates of targets. Furthermore, compared to the non-experts, the experts had faster information processing as indicated by earlier P3 peak latencies in an AB period, more attentional resources distributed to targets as indicated by stronger P3 amplitudes, and a more flexible deployment of attentional resources. These findings suggest that experts were less prone to the AB effect. Thus, long-term ARSG experience is related to improvements in temporal VSA. The current findings support the benefit of video gaming experience on the development of VSA.

## Introduction

Over the past few decades, video gaming has become an increasingly popular entertainment medium worldwide. Action video gaming—a major genre of video gaming—requires players to stay alert to stimuli in the peripheral region of view while tracking multiple targets simultaneously, and to make decisions under time pressure (Green and Bavelier, 2003, 2012). The research has examined the influence of action video gaming experience on cognitive development. Although some studies did not find evidence supporting the benefit of action video gaming experience on cognitive development (van Ravenzwaaij et al., 2014; Hilgard et al., 2019), meta-analyses have revealed positive influence of action video gaming on cognitive development (Latham et al., 2013; Powers et al., 2013; Wang et al., 2016). For example, action video gaming experience can improve primary information processing [e.g., visual processing (Green and Bavelier, 2007), contrast sensitivity (Li et al., 2009), hand-eye coordination (Dale and Green, 2017a)] and higher-level cognitive functions [e.g., attention, visuospatial processing, working memory and executive control (Green and Bavelier, 2003, 2012; Green and Seitz, 2015)].

Behavioral research showed that action video gaming experience is related to improvements in cognitive abilities that are highly relevant to visual selective attention (VSA; Dye et al., 2009; Green et al., 2010; Hubert-Wallander et al., 2011). For example, action video gaming experts outperformed non-experts in tasks of detecting and tracking fast moving objects (Green and Bavelier, 2006b; Boot et al., 2008), identifying central and peripheral visual stimuli (Green and Bavelier, 2006a), feature search and conjunction search (Wu and Spence, 2013), flanker compatibility, enumeration, and useful field of view (Bavelier et al., 2012). Furthermore, action video gaming training was found to improve performance on a useful field of view task (Green and Bavelier, 2003, 2006a). The useful field of view is the visual area over which information can be extracted at a brief glance without eye or head movements (Ball et al., 2002). In addition, compared with non-experts, action video gaming experts had better perception thresholds and processing speeds (Schubert et al., 2015), visual sensitivity (Appelbaum et al., 2013), visual short-term memory storage (Colzato et al., 2013; Blacker et al., 2014), top-down guidance in visual search (Wu and Spence, 2013), spatial distribution of attention (Feng et al., 2007; West et al., 2008), and oculomotor control (West et al., 2013). These findings are likely due to the fact that VSA is essential for action video gaming, as it enables players to selectively concentrate on a discrete aspect of information while ignoring other perceivable information. Thus, superior VSA can optimize the usage of attentional resources, allowing for successful action video gaming (Dye et al., 2009).

Neuroscience research has also examined the effects of action video gaming experience on VSA. For example, moving distractors elicited less activation of the visual motion-sensitive areas related to the suppression process in action video gaming experts than in non-experts, suggesting that experts more efficiently allocate attentional resources and filter irrelevant information (Bavelier et al., 2012). Research also examined learning-related brain plasticity using electrophysiological methods, which allow for the examination of temporally sensitive indicators (Luck, 2014). For example, by recording attention-related modulations of steady-state visually evoked potentials to target stimuli, research found that action video gaming experts could efficiently suppress the distraction of irrelevant information (Mishra et al., 2011; Krishnan et al., 2012). Furthermore, action video gaming experts could allocate attentional resources to task-relevant stimuli more efficiently than non-experts, as indicated by induced N2pc (West et al., 2015)—an event related potential (ERP) component linked to VSA (Eimer, 1996; Woodman and Luck, 1999).

Using a useful field of view task, electroencephalography research found that action video gaming experts had superior VSA relative to non-experts, as indicated by greater P2 and P3 amplitudes (Wu et al., 2012). These EEG indicators are closely related to VSA, as P2 amplitudes indicate attentional selection and attentional control processes (Carretié and Iglesias, 1995; Fritzsche et al., 2011) and P3 amplitudes reflect the allocation of attentional resources (Isreal et al., 1980; Wickens et al., 1983). This study (Wu et al., 2012)—along with behavioral studies (Green and Bavelier, 2003; Green et al., 2010)—used a useful field of view test to reveal the association between action video gaming experience and the development of VSA. However, it should be noted that VSA consists of multiple interrelated, yet distinct abilities, including *spatial* VSA which enables one to detect the location of a moving object and *temporal* VSA which enables one to detect an item among a sequence of items presented rapidly and consecutively in a single location. The useful field of view task taxes primarily spatial VSA, since participants are asked to locate the target stimulus that appears unpredictably, but equally often, in one of 24 different peripheral locations in a typical useful field of view task (Feng et al., 2007; Sungur and Boduroglu, 2012). Temporal VSA is also critical for action video gaming, as action video gaming players must detect and identify multiple stimuli appearing successively and quickly at the same location on the screen in action video gaming. However, the relationship between action video gaming experience and a critical component of VSA (i.e., temporal VSA) still remains understudied.

In recent years, action real-time strategy gaming (ARSG), which includes both *action* and *strategy* elements (Dale and Green, 2017a, b; Dale et al., 2019), is becoming increasingly popular. ARSG requires not only attention and hand-eye coordination but also strategic decision-making abilities based on instant responses and team cooperation, just like traditional team sports (e.g., football, basketball). Because ARSG contains action mechanics, it may tax a set of cognitive systems required in action video gaming; therefore ARSG may benefit cognitive development just like action video gaming (Dale and Green, 2017a; Dale et al., 2019). Indeed, fMRI research suggests that, compared with ARSG non-experts, experts have increased functional connectivity between the attentional and sensorimotor networks (Gong et al., 2015) as well as superior functional integration between salience and central executive networks—two critical networks for VSA (Gong et al., 2016). ARSG is therefore a cognitively demanding entertainment medium, offering a new perspective on the neural basis of cognitive and learning-related plasticity (Gong et al., 2017, 2019a,b; Kowalczyk et al., 2018; Qiu et al., 2018).

This study aims to determine whether ARSG experts have better temporal VSA than non-experts. As ARSG requires players to rapidly filter relevant information from irrelevant information, quickly decide on a course of action, and then execute that action in real-time context (Dale and Green, 2017a), this study seeks to provide direct evidence in terms of this view. This study differs from previous research in two ways. First, unlike Wu et al. (2012) and Qiu et al. (2018), this study used an attentional blink (AB) task—a time-based VSA task. AB refers to a deficit in reporting a second target (T2) presented within 200–500 ms after the onset of a first target (T1) in a rapid serial visual presentation (RSVP) stream (Vogel et al., 1998), which was confirmed as a capacity-limited stage by previous empirical research (Marois et al., 2000; Sergent et al., 2005). Research proposed that the AB effect is related to the attentional resources demanded in the processing of T1. Supporting this propostion is the finding that the AB effect is substantially reduced when participants are instructed to ignore T1 (Vogel et al., 1998; Kranczioch et al., 2003; Sessa et al., 2007). Furthermore, the AB phenomenon may arise from the limitation of attentional resources (Chun and Potter, 1995; Vogel et al., 1998) and the allocation of the attentional resources (Shapiro et al., 2006; Colzato et al., 2007; Martens and Wyble, 2010). Thus, the processing of T1 reduces the attentional resources available for T2 processing especially when T2 appears before T1 is fully processed. Martens and Wyble (2010) proposed that the relationship between T1 and T2 during the AB period suggests a temporal limit for reallocating attentional resources from T1 to T2. Since AB reflects temporal VSA limitations, an AB test is typically used to examine temporal VSA (Green and Bavelier, 2003; Marois and Ivanoff, 2005; Martens et al., 2006b; Dux and Marois, 2009; Willems and Martens, 2016). Although the useful field of view and AB tasks may utilize shared attentional resources, they may be related to different underlying attentional mechanisms, with spatial VSA operating at an early (perpetual) level of processing and temporal VSA operating only after perception is complete, therefore reflecting a response-related (post-perceptual) level of processing (Vogel et al., 1998; Griffin et al., 2002). Thus, an examination of the relationship between ARSG experience and temporal VSA is important for any complete theory on ARSG-related brain plasticity.

Second, unlike behavioral research (e.g., Green and Bavelier, 2003; Dye and Bavelier, 2010), this study used ERP methods, thus allowing us to examine temporally sensitive indicators which are not readily observable at the behavioral level (Luck, 2014). Using behavioral methods, research found a smaller AB effect in action video gaming experts than non-experts (Green and Bavelier, 2003). For example, Green and colleagues found that action video gaming experts outperformed non-experts in an AB task (Green and Bavelier, 2003). Research also found that action video gaming experts have a faster recovery of attention after an AB task than non-experts (Dye and Bavelier, 2010). Furthermore, action video gaming training improved the performance of non-experts in an AB task (Oei and Patterson, 2013, 2015). The current study used both behavioral and ERP data, aiming to reveal the cognitive time course of temporal VSA during an AB task.

The current study examines the P3 componet—an ERP component that is elicited over parietal regions of the scalp (Pz) in the process of working memory and typically reaches its greatest amplitude between 300 and 600 ms after stimulus onset (Donchin and Coles, 1988; Polich, 2004; Sessa et al., 2007)—because P3 is a sensitive indicator to T2 consolidation (Sessa et al., 2007). In electrophysiological AB research, a distinct P3 amplitude is often observed when T2 is identified; however, P3 is typically not observable for a “blinked” (i.e., an incorrectly reported or missed) T2. Thus, the suppression of T2-elicited P3 amplitude during the AB period suggests that a failure to consolidate T2 into working memory may result into a failure to correctly report T2 (Vogel et al., 1998; Vogel and Luck, 2002; Martens et al., 2006a, b; Sessa et al., 2007). In addition, P3 amplitude tends to decrease when T2 detection is impaired (Dell’Acqua et al., 2003). Thus, the AB effect can be observed through: (1) a decrease in T2 accuracy; and (2) a decrease in the T2-elicited P3 amplitude during the AB period.

Researchers have proposed that the AB effect is related to the allocation of attentional resources, which can be reflected by the P3 amplitude induced by target perception (Martens et al., 2006a). For example, Martens et al. (2006a) examined whether the amplitude of the T1-evoked P3 is related to the correct detection of T2. They found that the amplitude of T1-elicited P3 reflected the amount of attentional resources allocated to T1 processing and consolidation. A larger amplitude of the P3 indicates that fewer attentional resources are available for a period between 200 and 500 ms for the processing and consolidation of T2, thereby leading to an AB effect (Martens et al., 2006a).

Notably, target-locked P3 contains multiple subcomponents including the frontocentral P3a and the posterior P3b, which may indicate different processes at different levels of processing (Verleger et al., 2014; Verleger and Śmigasiewicz, 2016). The current study used P3b in data analysis for two reasons. First, P3b is an optimal marker of T2 consolidation in an AB task, as research confirmed that the amplitude of the posterior P3b decreased and its latency postponed during the AB period (Dell’Acqua et al., 2015). Second, analyzing P3b data collected through the Pz electrode is an established data analysis method used in previous AB research (e.g., Martens et al., 2006a, b; Sessa et al., 2007).

This study administered both ARSG experts and non-experts an AB test, where the between-group comparisons allowed us to evaluate the long-term effect of ARSG experience on temporal VSA. We predicted that ARSG experts should outperform non-experts in the AB task, based on previous behavioral findings (Green and Bavelier, 2003; Dye and Bavelier, 2010). Furthermore, the between-group differences can be indicated by the P3 component. As noted above, the P3 component indicates the attentional processes in an AB task (Vogel et al., 1998; McArthur et al., 1999; Martens et al., 2006b) and reflects the allocation of attentional resources (Luck et al., 2000; Allison and Polich, 2008; Maclin et al., 2011) with its latency representing the speed of information processing (Duncan-Johnson and Donchin, 1982; Martens et al., 2006b) and its amplitude signifying the amount of attentional resources allocated to stimuli (Isreal et al., 1980; Wickens et al., 1983).

## Materials and Methods

### Participants

Participants were recruited following the established procedure used in previous research (Qiu et al., 2018; Gong et al., 2019b). A survey was given, prior to the current study, to a number of participants who were asked to report: (1) their League of Legends (LOL^1^) Expertise Ranking and gaming experience (in years); (2) their LOL ID which was used to verify their self-reported gaming experience and ranking information, since their LOL Expertise Ranking is provided by the LOL game—the ARSG program used in this study; and (3) their experience (in years) of playing games other than LOL, which was used to exclude multi-genre gamers to ensure LOL was the primary game genre for all the participants recruited in this study. Only the individuals who were identified as either LOL experts or non-experts were invited to participate in this study. The participants were 38 males, healthy undergraduate and graduate students of the University of Electronic Science and Technology of China (UESTC). Both LOL experts (*M* = 20.53, *SD* = 2.04; *n* = 19) and non-experts (*M* = 21.32, *SD* = 2.08; *n* = 19) were recruited. All participants were right-handed, reported having normal or corrected-to-normal vision, and had no history of neurological problems. Informed consent was obtained before the experiment, and the test was approved by the UESTC Ethics Board. To minimize participant bias, the participants were not informed of their group membership or the purpose of this study. Participants were paid ¥150 on completion of the study.

Group membership was defined based on both time- and skill-based criteria following the procedure used in Qiu et al.’s (2018) study. The experts had at least 2 years of LOL experience and were recognized as LOL masters according to their Expertise Ranking (the top 7% of players)—an objective, widely used method for calculating the relative skill levels of LOL players. The non-experts had less than 0.5 years LOL experience and were recognized as non-experts based on their rankings (the lowest 29.92–45.11% of players).

LOL was used in this study because it is a typical ARSG program, containing both action and strategy elements. Indeed, fMRI research found that, compared with LOL non-experts, experts had increased functional connectivity between the attentional and sensorimotor networks (Gong et al., 2015) as well as superior functional integration between salience and central executive networks—two critical networks for VSA (Gong et al., 2016). Furthermore, a recent electrophysiological study revealed that compared with LOL non-experts, experts showed superior spatial VSA using the useful field of view task (Qiu et al., 2018). These findings demonstrate that LOL experience enhances VSA; thus, LOL provides an important platform for us to examine the effect of ARSG experience on the development of VSA.

### Stimuli and Apparatus

The presentation of visual stimuli and the collection of EEG responses were controlled by E-prime 2.0 on a Windows XP computer with a 3.30-GHz processor, a graphics card with 60 Hz temporal resolution, and a 21.5-inch Acer K222HQL monitor screen. Stimuli were digits (except 1 and 0) and consonant letters (except Q and Y). All stimuli were displayed individually and sequentially at the center of the monitor screen in Arial font, 3 cm high and 2 cm wide, in white against a black background.

### Procedure

Participants were seated in a dimly lit, sound attenuated, and electrically shielded testing booth, with their head placed on a fixed chin rest 60 cm away from the monitor screen to ensure a constant viewing distance. An experiment consisted of a practice block (24 trials) and four experimental blocks (120 trials each), and only the four experimental blocks were included in data analyses. A 5-min break was given after each block. An experimental session lasted approximately 120 mins.

**Figure 1:**
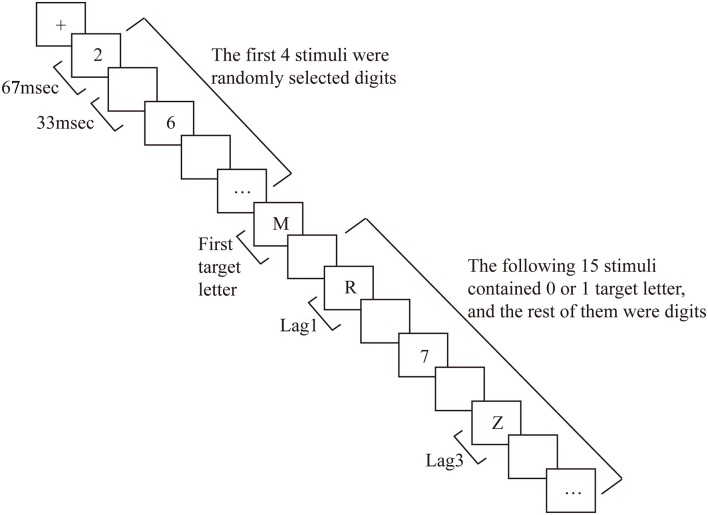
The attentional blink procedure used in this study.

Before each trial, a fixation cross was presented in the middle of the screen for 1,000 ms, followed by an RSVP stream consisting of 20 stimuli (including digits). Each item in the stream was presented for 67 ms, and successive items were separated by a blank interstimulus interval of 33 ms, yielding a presentation rate of 10 items per second (see Figure 1 for the experimental procedure). In 75% of the trials, two target letters were embedded in the stream (dual-target trials), in 25% of the trials, only one target letter was present (single-target trials). On dual- and single-target trials, T1 was presented as the fifth item in the stream. In dual-target trials, T2 was either the first, third, or eighth item following T1 (i.e., it was presented at lag1, 3, or 8, respectively). These specific lags were chosen based on the experimental procedure of previous research (Vogel et al., 1998; Martens et al., 2006b). For the ARSG non-experts, T2 is likely to be “blinked” (i.e., incorrectly reported) at lag3 (i.e., the AB period), whereas little or no reduction in T2 accuracy is usually observed at lag1 and 8 (Raymond et al., 1992; Chun and Potter, 1995; Vogel et al., 1998). Target letters were randomly selected with the constraint that T1 and T2 were always different letters. Digit distractors were randomly selected with the constraint that no single digit was presented twice consecutively.

To minimize possible eye blink and movement artifacts in the EEG at the end of the stream, the fixation cross reappeared in the middle of the screen for 1,000 ms at the end of the RSVP stream. Subsequently, participants were guided by a message at the bottom of the screen to type the letters they had seen using the corresponding keys on the computer keyboard. Participants were informed that they had enough time to make their responses to ensure that typing errors were not made. If a letter was not seen, the space bar was to be pressed instead. Thus, accuracy on single target trials means that participants correctly responded to T1 and correctly responded that there was no T2 (by pressing the space key). Participants were encouraged to make the T1 response before the T2 response on each trial although both the T1-T2 and T2-T1 orders were accepted. The results showed that the participants used the T1-T2 order almost exclusively in this study. The next trial started 1,000 ms after the participants made a response on the previous trial.

### EEG Recording and Analysis

The EEG data were collected on an EEG32-BT EEG amplifier (BORUIEN, China). Electrode position was based on the 10-20 system (Jasper, 1958). EEG was digitized with a sampling rate of 1,000 Hz. The impedance for all electrodes was kept below 5 kΩ, and all the data were online filtered with a 0.05–100 Hz bandpass filter. Scalp potentials were referenced to the average of left and right mastoids. To control for eye movement artifacts, horizontal and vertical electrooculograms (EOGs) were recorded from electrodes above the right eye and at the outer canthus of the left eye, respectively (Gratton et al., 1983). Off-line EEG analysis was performed according to a standard procedure using Brain Vision Analyzer Version 2.0.1 (Brain Products GmbH). The behavioral and EEG data were collected simultaneously.

All channels were re-referenced to “infinity” zero provided by the reference electrode standardization technique (REST) off-line (Yao, 2001). The ERPs were time locked to the onset of the target letter (T1 or T2) and were calculated relative to a 200 ms pre-stream baseline. EEG data were filtered with an IIR bandpass filter between 0.01 and 30 Hz and were corrected for EOG artifacts using ocular correction. To avoid eye movement and other artifacts, segments with maximum differences of values greater than 90 μV were excluded from additional analysis. ERPs for experimental conditions were obtained by averaging over trials.

The final data analyses included only the signals from Pz. As aforementioned, P3b is found to show topographical maximum at Pz (Verleger et al., 2014; Dell’Acqua et al., 2015; Verleger and Śmigasiewicz, 2016). In addition, electrophysiological AB research (i.e., Martens et al., 2006a, b; Sessa et al., 2007) typically uses Pz to analyze the P3 signal (P3b in specific according to Dell’Acqua et al., 2015). The electrodes other than Pz were used to obtain a clear scalp distribution of various components to ensure the accurate detection of the relevant waveform (P3). Because of the overlapping waveforms due to the temporal proximity of targets presented at lag1, analyses of the ERPs from dual-target trials were restricted to lag3 and 8. This study performed data analysis based on established procedures used in previous research (Martens et al., 2006b).

### Data Analysis

For the dual-target conditions, we conducted a 2 (group: experts, non-experts) × 3 (lag: 1, 3, 8) repeated measures ANOVA on the behavioral data, and a 2 (group: experts, non-experts) × 2 (lag: 3, 8) × 2 (target: T1, T2) repeated measures ANOVA on the ERP data. Bonferroni adjusted p-values were used for multiple comparisons.

## Results

### Behavioral Results

#### T1 Performance in the Single-Target Condition

For each participant, an accuracy rate was calculated across the four experimental blocks. An independent samples *t*-tests compared accuracy rates between groups. The results showed that accuracy rates were higher in the experts (*M* = 0.96, *SD* = 0.04) than in the non-experts (*M* = 0.90, *SD* = 0.09; *t*_(36)_ = 2.27, *p* < 0.05, *d* = 0.86).

#### T1 Performance in the Dual-Target Condition

**Figure 2:**
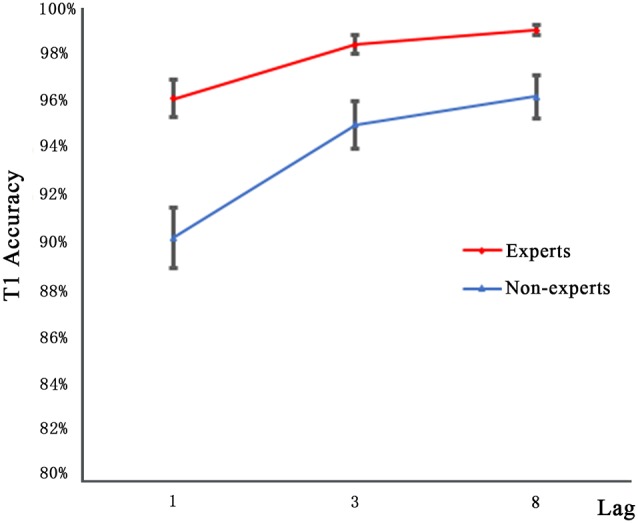
Mean accuracy for identifying T1 in the three lag conditions of experts and non-experts. Error bars stand for SE.

Figure 2 shows the T1 performance in the dual-target condition as a function of lag for both groups. Since, the accuracy data are mostly at the ceiling level, an arc sine transformation was performed to transform the accuracy data when they neared the ceiling level to ensure that the distribution of data matches the assumptions of the tests. A 2 × 3 repeated measures ANOVA with group (experts, non-experts) as the between-subjects variable and lag (1, 3, 8) as the within-subjects variable analyzed the accuracy rates. The main effect of group (*F*_(1,36)_ = 24.27, *p* < 0.001, $\eta_{\text{p}}^{2}$ = 0.40) was significant, suggesting that the experts had higher accuracy than the non-experts. We also found a main effect of lag (*F*_(2,72)_ = 30.40, *p* < 0.001, $\eta_{\text{p}}^{2}$ = 0.46); multiple comparisons with Bonferroni correction revealed that the accuracy rates of lag1 and lag3 as well as lag1 and lag8 differed significantly, yet the accuracy rates of lag3 and lag8 did not approach significance. Furthermore, the group × lag interaction was not significant (*F*_(2,72)_ = 0.87, *p* = 0.42).

#### T2 Performance in the Dual-Target Condition

**Figure 3:**
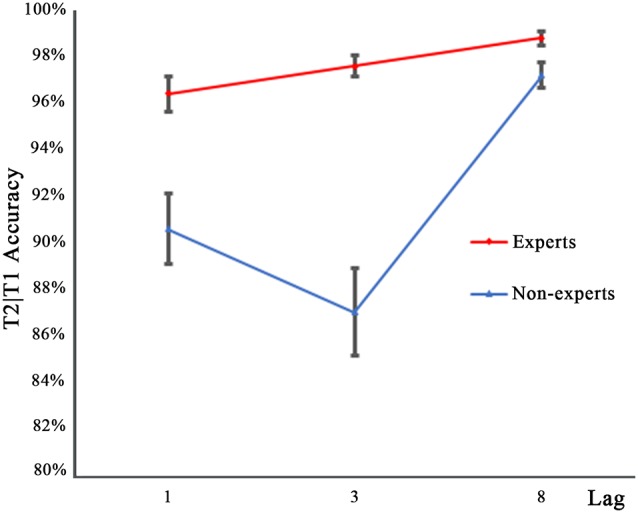
Mean accuracy for identifying T2 in the three lag conditions of experts and non-experts when T1 was correctly identified. Error bars stand for SE.

Figure 3 shows the performance for T2 on the trials where T1 was reported correctly (T2|T1), as a function of lag for either group. Similar to the T1 accuracy data, an arc sine transformation was performed to transform the accuracy data of T2. A 2 × 3 repeated measures ANOVA with group (experts, non-experts) as the between-subjects variable and lag (1, 3, 8) as the within-subjects variable analyzed the accuracy rates. The main effects of group (*F*_(1,36)_ = 44.91, *p* < 0.001, $\eta_{\text{p}}^{2}$ = 0.56) and lag (*F*_(2,72)_ = 30.63, *p* < 0.001, $\eta_{\text{p}}^{2}$ = 0.46) were significant. In addition, a significant group × lag interaction emerged (*F*_(2,72)_ = 7.68, *p* < 0.001, $\eta_{\text{p}}^{2}$ = 0.18). To decompose this interaction, separate one-sample repeated measure ANOVAs were conducted within each group. Bonferroni corrected *p* values were used in multiple repeated measures ANOVAs. Results showed that for the non-experts, T2 performance in the three lag conditions differed significantly (*F*_(2,36)_ = 27.89, *p* < 0.001, $\eta_{\text{p}}^{2}$ = 0.61), with lag3 registering the lowest detection rates and lag8 registering the highest. For the experts, there were significant differences across the three lag conditions (*F*_(2,36)_ = 6.79, *p* < 0.01, $\eta_{\text{p}}^{2}$ = 0.27); multiple comparisons with Bonferroni correction showed that the accuracy rates of lag1 and lag8 differed significantly, while the accuracy rates of lag1 and lag3 as well as lag3 and lag8 did *not* show significant differences, suggesting that the experts were less prone to the AB effect than the non-experts. *Post hoc* between-group comparisons conducted through separate independent samples *t*-tests showed that, compared with the non-experts, the experts had significantly higher accuracy rates for lag1 (*t*_(36)_ = 3.93, *p* < 0.001, *d* = 1.21) and lag3 (*t*_(36)_ = 7.17, *p* < 0.001, *d* = 2.43), but the accuracy rate of both groups did not reach significance for lag8 (*t*_(36)_ = 2.41, *p* = 0.02, *d* = 0.81; a significant cut off level of 0.017 was used).

### ERP Results

#### The Latency of P3

**Figure 4:**
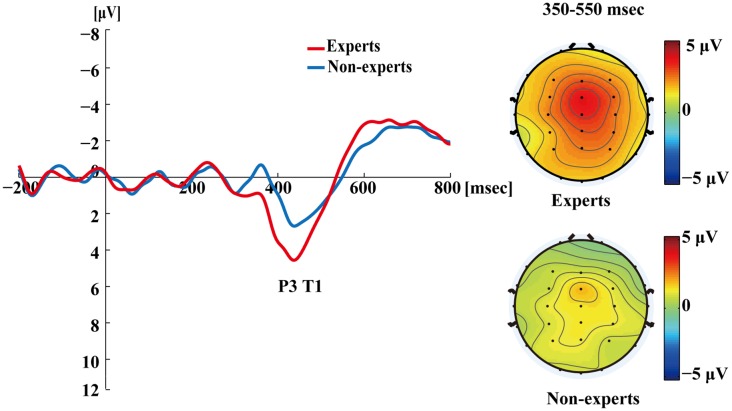
Grand averages of the mean activation at Pz of both experts and non-experts as a function of time for single-target trials. ERPs were time locked to the onset of T1. The scalp distribution of 350–550 ms time windows for single-target condition.

Figure 4 shows the ERP data for both groups in the single-target condition where T1 was correctly identified. A clear T1-related P3 response emerged. For each participant, the mean time required to process a target was calculated by subtracting the onset time of the target from the mean latency of the P3 peak evoked by it. An independent samples *t*-test showed that the mean latencies of P3 did not differ between the experts and the non-experts (*t*_(36)_ = 0.68, *p* = 0.50), suggesting that the experts and the non-experts did not differ in their processing speed for T1.

**Figure 5:**
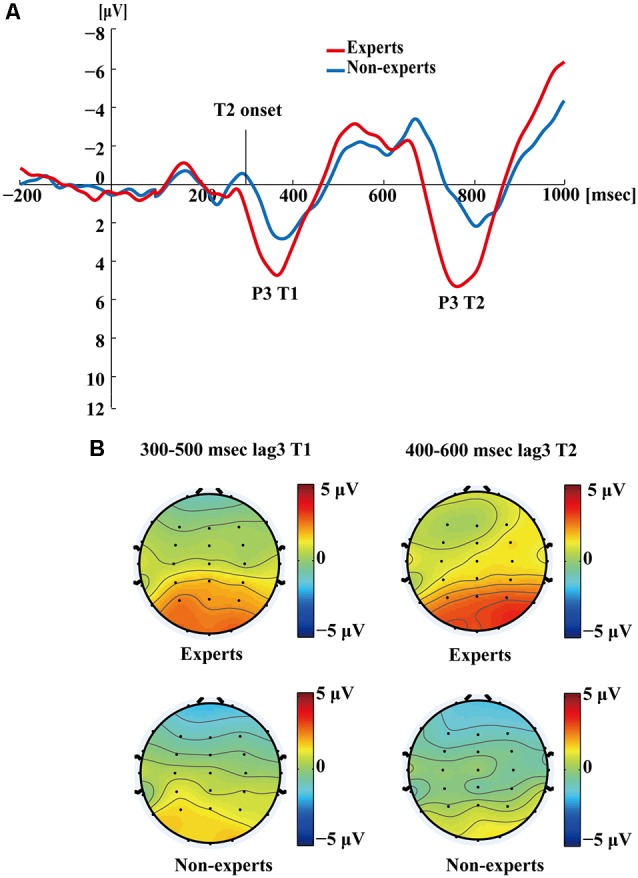
**(A)** Grand averages of the mean activation at Pz of experts and non-experts as a function of time for lag3 trials during which an attentional blink did not occur (nonblink trials). Owing to the onset proximity of T1 and T2, we drew the waves induced by both targets together. ERPs were time locked to the onset of T1. **(B)** The scalp distribution of 300–500 ms time windows for dual-target condition at lag3 for T1. The scalp distribution of 400–600 ms time windows for dual-target condition at lag3 for T2.

**Figure 6:**
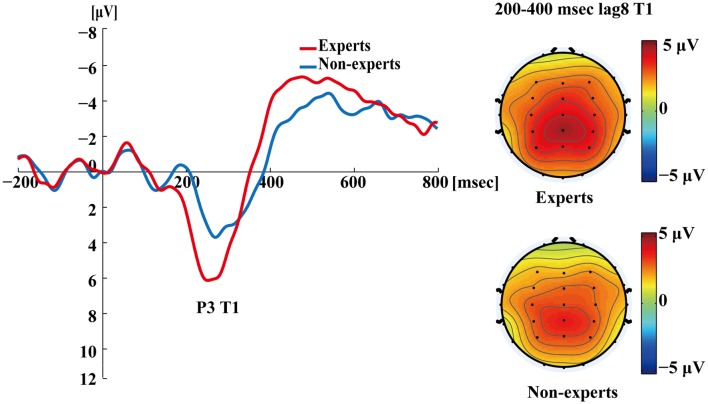
Grand averages of the mean activation at Pz of the experts and the non-experts as a function of time for lag8 trials of correct T1 identification. ERPs were time locked to the onset of T1. The scalp distribution of 200–400 ms time windows for dual-target condition at lag8 for T1.

**Figure 7:**
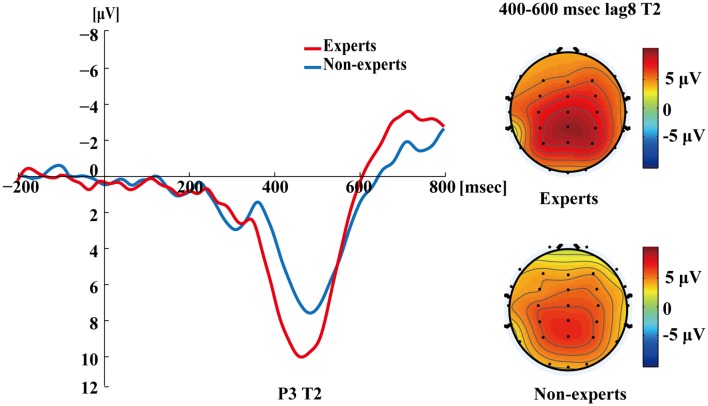
Grand averages of the mean activation at Pz of the experts and the non-experts as a function of time for lag8 trials of correct T2 identification. ERPs were time locked to the onset of T2. The scalp distribution of 400–600 ms time windows for dual-target condition at lag8 for T2.

Figure 5 shows the P3s induced by the two identified targets for both groups on nonblink trials at lag3. For T2, the experts appeared to have an earlier P3 peak than the non-experts, suggesting that the experts identified the targets faster, which may indicate an earlier processing of the relevant information. Figure 6 shows the P3s induced by identified T1 for both groups on nonblink trials at lag8. Figure 7 shows the P3s induced by identified T2 for both groups on nonblink trials at lag8.

The descriptive information of the peak latency of P3 (Supplementary Table S1) is available in the Supplementary Material. In the dual-target condition, a 2 × 2 × 2 repeated measures ANOVA with group as the between-subjects variable (experts, non-experts) and lag (3, 8) and target (T1, T2) as the within-subjects variables analyzed the peak latencies of P3. The main effects of group (*F*_(1,36)_ = 7.67, *p* < 0.01, $\eta_{\text{p}}^{2}$ = 0.18), lag (*F*_(1,36)_ = 325.86, *p* < 0.001, $\eta_{\text{p}}^{2}$ = 0.90), and target (*F*_(1,36)_ = 768.38, *p* < 0.001, $\eta_{\text{p}}^{2}$ = 0.96) were significant. In addition, a significant group × lag × target interaction emerged (*F*_(1,36)_ = 18.98, *p* < 0.001, $\eta_{\text{p}}^{2}$ = 0.35). To decompose the three-way interaction, a 2 (experts, non-experts) × 2 (T1, T2) repeated measures ANOVA was conducted for each lag condition. For the lag3 condition, a significant group × target interaction emerged (*F*_(1,36)_ = 12.66, *p* < 0.001, $\eta_{\text{p}}^{2}$ = 0.26). *Post hoc* between-group comparisons conducted through separate independent samples *t*-tests showed that the experts and non-experts had similar peak latencies of P3 induced by T1 (*t*_(36)_ = 0.17, *p* = 0.87). However, compared to the non-experts, the experts had significantly earlier P3 peaks induced by T2 (*t*_(36)_ = 8.19, *p* < 0.001, *d* = 2.66). For the lag8 condition, however, the 2 (experts, non-experts) × 2 (T1, T2) repeated measures ANOVA did not show a significant main effect of group (*F*_(1,36)_ = 2.31, *p* = 0.14) or a group × target interaction (*F*_(1,36)_ = 0.21, *p* = 0.65), suggesting P3 peaks did not differ between groups for the lag8 condition. See Supplementary Material for data analysis using the 50% area latency of P3, which generated a pattern of results similar to the results reported above.

#### The Amplitude of P3

Figures 4–7 showed that, the amplitudes of P3 were larger in the experts than in the non-experts, suggesting that the experts distributed more attentional resources to targets as compared to non-experts.

Since, P3 latencies appeared at different times between conditions based on the grand averages, we examined the P3 amplitude using different time segments based on the established method used in Martens et al. (2006a). This data processing method was proposed by Martens et al. (2006a), where the P3 amplitude was computed by identifying specific time windows (based on visual inspection of the grand average), from which the mean ERP signal was calculated by averaging the voltage of each individual data point within the specified window. For single-target trials, P3 amplitudes evoked by T1 were calculated as the mean amplitudes of the waveform from 350 to 550 ms post-T1 onset; for dual-target trials at lag3, P3 amplitudes evoked by T1 were calculated as the mean amplitudes of the waveform from 300 to 500 ms post-T1 onset; for dual-target trials at lag3, P3 amplitudes evoked by T2 were calculated as the mean amplitudes of the waveform from 400 to 600 ms post-T2 onset; for dual-target trials at lag8, P3 amplitudes evoked by T1 were calculated as the mean amplitudes of the waveform from 200 to 400 ms post-T1 onset; for dual-target trials at lag8, P3 amplitudes evoked by T2 were calculated as the mean amplitudes of the waveform from 400 to 600 ms post-T2 onset. Furthermore, P3 amplitude could also be computed using the peak amplitude of the average waveforms by visual inspection of each subject’s data. This method can reduce the between-subjects variance of P3 latency (Martens et al., 2006a; see Supplementary Material for results of the peak amplitude of P3).

In the single-target condition, an independent samples *t*-test showed that the experts had greater P3 amplitudes than the non-experts (*t*_(36)_ = 4.30, *p* < 0.001, *d* = 1.39). In the dual-target condition, a 2 × 2 × 2 repeated measures ANOVA with group (experts, non-experts) as the between-subjects variable and lag (3, 8) and target (T1, T2) as the within-subjects variables analyzed the mean amplitudes of P3. The main effects of group (*F*_(1,36)_ = 8.56, *p* < 0.01, $\eta_{\text{p}}^{2}$ = 0.19), lag (*F*_(1,36)_ = 131.72, *p* < 0.001, $\eta_{\text{p}}^{2}$ = 0.79), and target (*F*_(1,36)_ = 21.78, *p* < 0.001, $\eta_{\text{p}}^{2}$ = 0.38) were significant. In addition, a significant group × target interaction emerged (*F*_(1,36)_ = 5.64, *p* < 0.05, $\eta_{\text{p}}^{2}$ = 0.14). *Post hoc* between-group comparisons were conducted through separate independent samples *t*-tests. The results showed that the P3 mean amplitudes for T2 were greater in the experts than in the non-experts (*t*_(36)_ = 3.63, *p* < 0.001, *d* = 1.18), but the P3 mean amplitudes for T1 did not differ between groups (*t*_(36)_ = 1.41, *p* = 0.17). Furthermore, none of the other interactions were significant (*p*’s > 0.05).

Then, planned paired-sample *t*-tests were conducted within each group, focusing on the AB period. The results showed that at lag3, the P3 mean amplitudes of T1 and T2 did not differ significantly within the experts (*t*_(18)_ = 0.89, *p* = 0.39). However, for the non-experts, larger mean P3 amplitudes were evoked by T1 than by T2 (*t*_(18)_ = 2.29, *p* < 0.05, *d* = 0.46). See Supplementary Material for the analyses of the peak amplitude of P3, which generated a pattern of results similar to the analyses of the P3 mean amplitudes.

## Discussion

Using both behavioral and electrophysiological measures, this study examined the influence of ARSG experience on the development of temporal VSA—a major component of VSA that still remains understudied. Both ARSG experts and non-experts completed an AB task, which is typically used to examine temporal VSA. The behavioral data showed that the experts had higher identification rates than the non-experts. The electrophysiological data showed that compared to the non-experts, the experts had faster information processing as indicated by earlier P3 peak latencies during an AB period and more attentional resources distributed to targets as indicated by greater P3 amplitudes. Furthermore, the experts were less prone to the AB effect, since the T2 identification rates decreased at lag3 in the non-experts but not in the experts. Thus, this study showed that the experts had better temporal VSA than the non-experts in an AB task, consistent with the previous findings based on behavioral measures (Green and Bavelier, 2003; Oei and Patterson, 2013, 2015; but see Boot et al., 2008 for counter-arguments). Research has proposed that long-term action video gaming experience may improve the performance on accuracy-based tasks like the AB task by increasing the attentional resources and/or enhancing the ability to allocate those resources across time (Dye et al., 2009). By showing that ARSG experience is related to the development of VSA, we found that this proposition also applies to ARSG in this study, which may be due to the fact that ARSG contains “action” mechanics (Dale and Green, 2017a, b; Dale et al., 2019).

The ERP results showed faster information processing in the experts than the non-experts, as indexed by the experts’ earlier P3 peak latencies during the AB period. Since the latency of P3 reflects the speed of information processing (Duncan-Johnson and Donchin, 1982; Martens et al., 2006b), the experts’ earlier P3 latencies may indicate that they can detect targets more efficiently than the non-experts, which ensures their rapid response to the targets. This study also found that the experts had earlier P3 peaks than the non-experts at lag3, revealing the experts’ faster processing of the targets during the AB period. Furthermore, since P3 latencies are often postponed due to attentional limitations in an AB paradigm (Sessa et al., 2007), the non-experts’ delayed P3 latencies observed in this study confirm that they were more prone to an AB effect and had greater attentional limitations than the experts.

Furthermore, this study found that the experts distributed more attentional resources to targets than the non-experts as indexed by the experts’ greater P3 amplitudes during the AB period, since P3 amplitude indicates the amount of attentional resources allocated to stimuli (Isreal et al., 1980; Wickens et al., 1983). Using electrophysiological methods, research found that AB arises from not only the limitation of attentional resources (Chun and Potter, 1995; Vogel et al., 1998), but also the allocation of the limited attentional resources (Shapiro et al., 2006; Colzato et al., 2007; Martens and Wyble, 2010). A larger P3 amplitude induced by T2 indicates a greater distribution of attentional resources to T2, which can decrease the likelihood of the occurrence of AB (Martens et al., 2006a). Indeed, this study found that the P3 amplitudes induced by T2 at both lag3 and lag8 were greater in the experts than the non-experts.

Research showed that in an AB task, a greater magnitude of the P3 elicited by T1 indicates that less attentional resources are allocated for the processing of T2, thus increasing the likelihood of the occurrence of AB (McArthur et al., 1999; Fell et al., 2002; Shapiro et al., 2006). This study found that during the AB period, the amplitudes of P3 induced by T1 were greater than those induced by T2 within the non-experts, but the amplitudes of P3 did *not* differ between T1 and T2 within the experts. These findings suggest that the non-experts distributed more attentional resources to T1 than T2, but experts distributed attentional resources similarly between T1 and T2. This may contribute to the between-group differences in the AB task observed in this study, and suggest that the experts can allocate attentional resources more flexibly than the non-experts (Shapiro et al., 2006; Colzato et al., 2007).

Dux and Marois (2009) suggested that AB arises from the competition between targets (T1, T2) for attentional resources at short T1-T2 lags (Dux and Marois, 2009). The attentional demand of T1 for working memory encoding (Vogel et al., 1998), episodic registration (Wyble et al., 2009), and response selection (Jolicoeur, 1999) prevents attentional resources being distributed to T2 for the enhancement of target representations (Vogel and Luck, 2002) and the inhibition of distractors. Detecting a target in the RSVP stream triggers an attentional episode, which leads to the enhancement of the representations of both the target stimulus and the stimulus that immediately follows. However, all these stimuli are processed in the same attentional window competing for attentional resources to be admitted to higher stages of processing. Thus, since attentional resources are limited in an individual, a greater distribution of attentional resources to T1 (due to either its perceptual salience or earlier presentation order in the RSVP stream) is inherently related to a smaller distribution of attentional resources to T2, leading to the failure of reporting T2. According to Dux and Marois (2009), experts could deploy attentional resources more efficiently than non-experts during the AB period, thus ensuring enough attentional resources for multiple processes mentioned above. Thus, AB is less likely to occur in the experts than in the non-experts.

Notably, there are alternative explanations to the occurrence of AB besides the temporal limitations of attentional resources. For example, research suggested that AB might be a result of a strategy to avoid confusing the order of events (Wyble et al., 2009, 2011). Furthermore, paradoxically, there is evidence showing that distracting participants’ attention from the RSVP task (by introducing an additional task into the RSVP task—e.g., listening to music, thinking about holiday plans, discriminating the presence of a red dot during the AB task) may decrease the magnitude of the AB effect (Olivers and Nieuwenhuis, 2005, 2006; Olivers, 2007; Taatgen et al., 2009). In addition, researchers proposed that concurrency benefits in the AB was linked to shifts in decision criteria (Lapointe-Goupil et al., 2011). However, the replicability of these findings needs further examination (i.e., see Footnote 1 in Olivers and Nieuwenhuis, 2006, which noted that attempts to replicate the result of listening to music had failed). In addition, introducing an additional task in the RSVP task may result in a more shallow level of stimulus processing (Willems and Martens, 2016). Nevertheless, although it is a widely tested theoretical model that AB occurs because of the temporal limitations of attentional resources, the mechanism of the occurrence of AB still demands further investigation.

Interestingly, this study found that the T1-evoked P3 has a frontocentral maximum (i.e., P3a) in single-target condition (see Figure 4) but a posterior maximum (i.e., P3b) in dual-target conditions (Figures 5, 6). Perhaps, this is related to the experimental design that there was only one target among distractors in the RSVP in the single-target condition, which might have enhanced the salience of the target and therefore made it relatively easy to detect. Nevertheless, P3a may reflect the deployment of attention for detection of *contextually salient* information presented amongst distracting stimuli (Polich, 2007). Notably, Vogel et al. (1998) also observed P3 waveforms at the central midline electrode sites in the single target condition (see Figure 9 in Vogel et al., 1998), which might be P3a according to Verleger et al. (2014) and Dell’Acqua et al. (2015). Research suggests that AB engages a frontoparietal attention circuit, which is observable through P3a and P3b induced by target identification (Sergent et al., 2005; Dell’Acqua et al., 2015). Nevertheless, few studies have investigated the target-evoked P3 in the single-target condition where only one target is presented. Future studies should determine whether the single-target condition systematically differ from dual-target conditions in the AB task.

This study used the P3 amplitude as a measure of attentional resources, which is a typical data analysis procedure used in previous electrophysiological AB research (i.e., Sergent et al., 2005; Martens et al., 2006a). However, it should be noted that the use of P3b amplitude as a measure of attentional resources has been found to be ambiguous. For example, Kok (2001) found that P3 amplitudes may decrease in difficult tasks. Thus, perhaps, in the current study, the experts had larger P3s than the non-experts simply because this task was less difficult for the experts than for the non-experts. The fact that this study only has one cross-sectional experiment does not allow us to rule out this possibility. Future research should further examine the mechanism of the P3 amplitudes in the AB context.

Is video gaming experience related to the development of VSA in general or merely *computer-screen-related* VSA? The current study does not allow us to evaluate this question since this study only used a computer-screen-based VSA task. However, using the useful field of view task, Green and Bavelier (2003) found that playing video gaming leads to detectable effects on new tasks. Anguera et al. (2013) found that those playing 15-h video gaming showed not only improvement in all practiced video games, but also enhancements in two visuospatial working memory tasks as well as the episodic memory and short-term memory tasks. Furthermore, gains in some working memory and episodic memory tasks were maintained during a 3-month follow-up period (Anguera et al., 2013). Thus, it appears that video gaming experience is related to the development of VSA in general.

Nonetheless, the correlational nature of this study precludes drawing causal conclusions. The current findings support the relationship between action video gaming/ARSG experience and VSA development previously observed by both behavioral and electrophysiological research (Green and Bavelier, 2003; Oei and Patterson, 2013, 2015; Qiu et al., 2018). This study showed that ARSG experience is related to the development of temporal VSA—a major component of VSA previously unexamined. In addition, using electrophysiological measures, this study revealed the cognitive time course of AB in ARSG experts and non-experts.

## Data Availability Statement

The datasets generated for this study are available on request to the corresponding author.

## Ethics Statement

The experimental protocols were approved by the ethics research committee of the University of Electronic Science and Technology of China (UESTC), and were performed in accordance with ethical standards outlined by the Declaration of Helsinki. Informed consents were obtained from all subjects.

## Author Contributions

DG, HL and TL proposed the framework of the study. XG, YY, JX and DJ designed the experiment. XG, XZ, SY and XT conducted the experiment. DG, XG and WM wrote the manuscript. XG, RC, NQ and LD analyzed the data. DG, HL and WM provided important revision for the manuscript.

## Conflict of Interest

The authors declare that the research was conducted in the absence of any commercial or financial relationships that could be construed as a potential conflict of interest.

## Footnotes

^1^The LOL, a video game that requires players to cooperate with teammates to destroy the opposing team’s towers, is a typical ARSG game, which is also commonly referred to as the Multiplayer Online Battle Arena (MOBA) genre. The LOL consists of three game modes: Summoner’s Rift (Ranked Matchmaking), Twisted Treeline, and Howling Abyss (Normal Matchmaking). Players’ gaming experience and expertise are indicated by their Ranked Matchmaking level which is calculated based on the Elo rating system—a method for calculating the relative skill levels of players in competitor-versus-competitor games. The experts and non-experts can be defined based on the Elo rating of their Ranked Matchmaking level, which is available from an online inventory (http://www.lol91.com/duanweibilv.html). The Elo rating system has multiple stages—Bronze, Silver, Gold, Platinum, Diamond, Master, and Challenger—across which the expertise level increases sequentially. Each stage has five phases ranging from V to I. In the Ranked Matchmaking mode, players who win (or lose) a game will gain (or lose) a certain amount of points depending on the champion’s performance. Wining each 100 points activates the promotion competition, the successful completion of which promotes the player to the next stage or level. The successful maintenance of points requires one to play LOL on a regular and frequent basis.
