# Supplementary material for: Action Real-Time Strategy Gaming Experience Related to Increased Attentional Resources: An Attentional Blink Study
Source: Front Hum Neurosci. 2020 Apr 10;14:101. doi: 10.3389/fnhum.2020.00101 (PMC7163005; doi:10.3389/fnhum.2020.00101)
Supplement: Supplementary file 1 [file Table_1.DOCX]

Supplementary Material

- Supplementary Results 1-2
- Supplementary Tables 1-2

**Supplementary Result 1. The 50% area latency of P3**

We also calculated the 50% area latencies which were quantified as the time point that bisected the area under the P3 waves. In different conditions, the time windows used for this P3 latency measure were the same as those used for measuring the mean P3 amplitudes.

In the single-target condition, an independent samples *t* test showed that the latencies of P3 did not differ between the experts and the non-experts [*t*_(36)_ = 1.17, *p* = 0.25].

The descriptive information of the 50% area latency of P3 could be found in Table 2. In the dual-target condition, a 2 × 2 × 2 repeated measures ANOVA with group as the between-subjects variable (experts, non-experts) and lag (3, 8) and target (T1, T2) as the within-subjects variables analyzed the latencies of P3. The main effects of group [*F*_(1,36)_ = 26.29, *p* < 0.001,$\eta_{p}^{2}$ = 0.42], lag [*F*_(1,36)_ = 215.75, *p* < 0.001, $\eta_{p}^{2}$ = 0.86], and target [*F*_(1,36)_ = 1842.05, *p* < 0.001, $\eta_{p}^{2}$ = 0.98] were significant. In addition, a significant group × lag × target interaction emerged [*F*_(1,36)_ = 7.71, *p* < 0.01, $\eta_{p}^{2}$ = 0.18]. To decompose the three-way interaction, a 2 (experts, non-experts) × 2 (T1, T2) repeated measures ANOVA was conducted for each lag condition. For the lag3 condition, a significant group × target interaction emerged [*F*_(1,36)_ = 4.54, *p* < 0.05, $\eta_{p}^{2}$ = 0.11]. Post-hoc between-group comparisons conducted through separate independent samples *t* tests showed that the experts and non-experts had similar P3 latencies induced by T1 [*t*_(36)_ = 2.66, *p* = 0.012, *d* = 0.86]. However, compared to the non-experts, the experts had significantly earlier P3 latencies induced by T2 [*t*_(36)_ = 6.14, *p* < 0.001, *d* = 1.99]. For the lag8 condition the 2 (experts, non-experts) × 2 (T1, T2) repeated measures ANOVA did not show a group × target interaction [*F*_(1,36)_ = 0.21, *p* = 0.65].

**Supplementary Result 2. The peak amplitude of P3**

We also computed P3 amplitudes as the amplitudes of the peak of the response under different conditions.

In the single-target condition, an independent samples *t* test showed that the experts had greater P3 amplitudes than the non-experts [*t*_(36)_ = 2.72, *p* < 0.01, *d* = 0.88]. In the dual-target condition, a 2 × 2 × 2 repeated measures ANOVA with group (experts, non-experts) as the between-subjects variable and lag (3, 8) and target (T1, T2) as the within-subjects variables analyzed the peak amplitudes of P3. The main effects of group [*F*_(1,36)_ = 10.38, *p* < 0.01, $\eta_{p}^{2}$ = 0.22], lag [*F*_(1,36)_ = 85.75, *p* < 0.001, $\eta_{p}^{2}$ = 0.70], and target [*F*_(1,36)_ = 19.52, *p* < 0.001, $\eta_{p}^{2}$ = 0.35] were significant. In addition, a significant group × target interaction emerged [*F*_(1,36)_ = 6.92, *p* < 0.05, $\eta_{p}^{2}$ = 0.16]. Post-hoc between-group comparisons were conducted through separate independent samples *t* tests. Results showed that the P3 peak amplitudes for T2 were greater in the experts than in the non-experts [*t*_(36)_ = 3.85, *p* < 0.001, *d* = 1.25], but the P3 peak amplitudes for T1 did not differ between groups [*t*_(36)_ = 1.81, *p* = 0.08]. Furthermore, none of the other interactions were significant (*p’s* > 0.05).

Then, planned paired-sample *t* tests were conducted within each group, focusing on the AB period. Results showed that at lag3, the P3 peak amplitudes of T1 and T2 did not differ significantly within the experts [*t*_(18)_ = 1.00, *p* = 0.33]. However, for the non-experts, greater P3 peak amplitudes were evoked by T1 than by T2 [*t*_(18)_ = 4.35, *p* < 0.001, *d* = 0.81].

**Supplementary Table 1**

| Group | | T1 | | T2 | |
| --- | --- | --- | --- | --- | --- |
|  |  | lag3 | lag8 | lag3 | lag8 |
|  | experts | 360.16/24.29  (19) | 255.26/25.71  (19) | 455.63/23.36  (19) | 465.42/31.01  (19) |
|  | non-experts | 362.63/59.60  (19) | 273.26/49.15  (19) | 518.58/23.99  (19) | 478.53/31.92  (19) |

Table S1. The descriptive information of P3 peak latency (M/SD). (number of subjects in Parentheses)

**Supplementary Table 2**

| Group | | T1 | | T2 | |
| --- | --- | --- | --- | --- | --- |
|  |  | lag3 | lag8 | lag3 | lag8 |
|  | experts | 375.05/18.18  (19) | 280.58/16.58  (19) | 473.47/21.67  (19) | 483.37/18.04  (19) |
|  | non-experts | 399.11/35.00  (19) | 295.05/23.09  (19) | 518.42/23.41  (19) | 494.74/16.05  (19) |

Table S2. The descriptive information of the 50% area latency of P3 (M/SD). (number of subjects in Parentheses)
